# Rat Grimace Scale as a Method to Evaluate Animal Welfare, Nociception, and Quality of the Euthanasia Method of Wistar Rats

**DOI:** 10.3390/ani13203161

**Published:** 2023-10-10

**Authors:** Adriana Domínguez-Oliva, Adriana Olmos-Hernández, Ismael Hernández-Ávalos, Hugo Lecona-Butrón, Patricia Mora-Medina, Daniel Mota-Rojas

**Affiliations:** 1 Master in Science Program “Maestría en Ciencias Agropecuarias”, Universidad Autónoma Metropolitana, Xochimilco Campus, Mexico City 04960, Mexico; 2 Neurophysiology of Pain, Behavior and Assessment of Welfare in Domestic Animals, DPAA, Universidad Autónoma Metropolitana (UAM), Mexico City 04960, Mexico; 3 Division of Biotechnology—Bioterio and Experimental Surgery, Instituto Nacional de Rehabilitación-Luis Guillermo Ibarra Ibarra (INR-LGII), Mexico City 14389, Mexico; 4 Clinical Pharmacology and Veterinary Anesthesia, Biological Sciences Department, Facultad de Estudios Superiores Cuautitlán, Universidad Nacional Autónoma de México, Cuautitlán Izcalli 54714, Mexico; 5 Facultad de Estudios Superiores Cuautitlán, Universidad Nacional Autónoma de México, Cuautitlán Izcalli 54714, Mexico

**Keywords:** facial expression, decapitation, CO_2_ exposure, isoflurane, ketamine + xylazine

## Abstract

**Simple Summary:**

This study aimed to evaluate the pain associated with six methods of euthanasia: pentobarbital, CO_2_, decapitation, isoflurane, ketamine + xylazine, and ketamine + CO_2_ in Wistar rats, by applying the Rat Grimace Scale (RGS), comparing the scores, and determining the method with the highest score that might indicate pain in laboratory rodents. According to the results, during the application of the euthanasia method, the intraperitoneal administration of ketamine + xylazine and decapitation caused the highest RGS scores (0.6 ± 0.26 and 0.6 ± 0.16, respectively) (*p* < 0.0001), while after the application of the euthanasia methods, CO_2_ and isoflurane recorded the highest scores (*p* < 0.0001) (0.9 ± 0.18 and 1.2 ± 0.20, respectively). The results might indicate that injection and guillotine use could cause short-term pain in rodents, while high isoflurane scores could be associated with nociception/pain or the myorelaxant properties of the drug. Further research is needed to establish a comprehensive study of pain during euthanasia, where the RGS could be used minding the limitations that anesthetics might have on facial expression.

**Abstract:**

Refinement of experimental procedures in animal research has the objective of preventing and minimizing pain/distress in animals, including the euthanasia period. This study aimed to evaluate pain associated with six methods of euthanasia in Wistar rats (injectable, inhalational, and physical), by applying the Rat Grimace Scale (RGS), comparing the scores, and determining the method with the highest score that might indicate pain for laboratory rodents. Sixty adult male and female Wistar rats were used and assigned to six treatments: pentobarbital, CO_2_, decapitation, isoflurane, ketamine + xylazine, and ketamine + CO_2_. Video recording to assess the RGS scores was performed in four events: basal: 24 h before the procedure; Ti_1_: three minutes before the procedure; Ti_2_: during the application of the euthanasia method; and Ti_3_: immediately after the application until LORR. The main findings of this study showed that, during Ti_2_, decapitation and ketamine + xylazine had the highest scores (0.6 ± 0.26 and 0.6 ± 0.16, respectively) (*p* < 0.0001), while at Ti_3_, CO_2_ (0.9 ± 0.18) and isoflurane (1.2 ± 0.20) recorded the highest scores (*p* < 0.0001). According to the present results, decapitation and ketamine + xylazine elicited short-term acute pain, possibly due to tissue damage caused by both methods (injection and guillotine). In contrast, isoflurane’s RGS scores recorded during Ti_3_ might be associated with nociception/pain due to the pungency of the drug or to the pharmacological muscle relaxant effect of isoflurane. Further research is needed to establish a comprehensive study of pain during euthanasia, where RGS could be used minding the limitations that anesthetics might have on facial expression.

## 1. Introduction

Refinement is a principle proposed by the National Center for the Replacement, Refinement, and Reduction (NC3Rs) to prevent or minimize pain, suffering, or distress in research animals [1,2]. Currently, one of the most controversial topics regarding the use of laboratory rodents is the potential pain that animals might experience due to the application of injectable/inhalational drugs and the physical methods that can also be used for euthanasia [3,4]. Therefore, refinement does not only apply during the animals’ lives but also needs to be extended to the euthanasia period to prevent pain.

Nociception, known as the “neural processes of encoding and processing noxious stimulus” is the first step necessary to transmit nervous signaling from peripheral nociceptors to spinal and cerebral centers [5]. Pain is referred to as an “unpleasant sensory and emotional experience associated with, or resembling that associated with, actual or potential tissue damage”, in which by definition, the verbal inability to communicate pain does not negate that non-human animals might experience it [6]. Therefore, pain is the conscious perception of nociceptive signaling [5]. When an animal perceives pain, a cascade of physiological, endocrine, behavioral, and immune responses are triggered, irrupting its homeostasis [7]. Preventing pain in laboratory rodents is not only a scientific, legal, moral, and ethical duty but can also help to minimize variability and low reproducibility of results due to the mentioned alterations [8].

During the application of euthanasia, pain might be present due to the method or the properties of the drug. For example, injectable agents such as sodium pentobarbital and ketamine have basic or acid pH, respectively, that can cause pain due to tissue irritation after intraperitoneal (IP) administration [9]. Contrarily, other studies have not found pain-related behaviors (e.g., abdominal writhing) after IP injection of pentobarbital [10]. Physical methods such as decapitation are considered aesthetically unpleasant and the possibility of feeling conscious pain is under debate [11]. Several authors have reported that cerebral activity is maintained for 2.7–40 s after decapitation [12] and that the presence of low voltage, fast electroencephalographic (EEG) activity is compatible with conscious awareness of pain [13]. Nonetheless, due to the short period of consciousness and the possibility that the EEG indices represent cerebral cortex activity and not nociception, decapitation is still considered a humane euthanasia method [14,15]. Regarding inhalant methods, CO_2_ is a common euthanasia method, but some studies refer to the potential pain and activation of acid-sensitive channels (ASIC) due to carbonic acid formation when CO_2_ is combined with water in the nasal or ocular mucosa [16]. Moreover, although isoflurane is considered an alternative to CO_2_, its moderate pungency might be aversive and can cause discomfort in rodents [9,17].

Due to the potential nociceptive activation and/or pain that laboratory rodents might perceive during an experimental protocol, implementing tools for pain recognition is one of the essential steps for refining procedures. One of the most used methods is evaluating pain-related behaviors, such as writhing, back arching, twitching, stagger-fall, or lack of grooming and burrowing, among others [18]. Currently, studying pain facial expressions in domestic and wildlife species is a trend derived from Darwin’s studies regarding emotion and its association with facial expressions [19]. This has led to the development of “grimace scales”, scoring systems that categorize movements of facial muscles—called Facial Action Units (FAUs)—related to pain [20,21,22,23]. For rats, Sotocinal et al. [22] developed the Rat Grimace Scale (RGS), using as a basis the Mouse Grimace Scale (MGS), a validated tool that uses four FAUs to determine the pain level: 1. ear change; 2. orbital tightening; 3. nose/cheek flattening; and 4. whisker change.

On a scale of zero to two, zero = not present; one = moderately present; and two = obviously present, the RGS has been used to non-invasively evaluate surgical, visceral, orthopedic, and inflammatory pain [24]. It has been applied as a method to refine analgesic protocols or the efficacy of the analgesic drug [25,26], as well as in animal models of acute/chronic/neuropathic pain and to assess the effects of routine practices, such as drug administration or euthanasia procedures [25,27,28].

Animal research is committed to maintaining high standards of animal care and welfare. Recognizing and preventing pain is part of the proposed strategies to minimize the potential behavioral, physiological, and immune alterations that rodents might experience during an experimental protocol and euthanasia, also decreasing the confounding effects that pain might have on the research. The authors hypothesize that, regardless of the euthanasia method, the RGS will be able to distinguish changes in rats FAU due to the application of the method. Therefore, this study aimed to evaluate pain associated with six methods of euthanasia in Wistar rats (injectable, inhalational, and physical), by applying the RGS and comparing the scores. Additionally, we studied the correlation between each euthanasia method with a specific FAU, to determine the method with the highest score that might indicate pain for laboratory rodents.

## 2. Material and Methods

### 2.1. Study Animals and Housing Standards

The present study was performed in the Animal Facility and Experimental Surgery service from the Biotechnological Research Sub-department of the Instituto Nacional de Rehabilitación-Luis Guillermo Ibarra Ibarra, Mexico City, Mexico.

Sixty adult Wistar rats (*R. norvegicus*), thirty male and thirty female, were obtained from the Center for Research and Advanced Studies at the National Polytechnic Institute (CINVESTAV-IPN) (average weight of 311 ± 62 g at 8–10 weeks old).

In accordance with the 3Rs principles [29], reduction in the use of laboratory animals was encouraged by reusing rats coming from control groups of concluded behavioral tests (e.g., balance beam or maze tests). A physical exam considering body weight, posture, level of consciousness, secretions, the color of the mucosa, sneezing, and species-specific behavioral repertoire was performed to select healthy animals. Rats that underwent invasive procedures, had residual drugs, or signs of disease, stress, and pain, or were pregnant were excluded. It is important to mention that the selected rats were also used as a part of a recently published study about thermal imaging during euthanasia [30], performing infrared and RGS assessments simultaneously.

Rats were placed in groups of five animals per cage. Standard polycarbonate cages for rats were used (47 × 36 × 21 cm), with wood shavings as bedding (Aspen, Nepco, Warrensburg, NY, USA) and without enrichment. A controlled temperature inside the housing room was set to an average of 23.2 ± 0.5 °C and 48% relative humidity, maintaining a 12 h day–night cycle (lights on between 0500 h and 1700 h). The rats had ad libitum access to food (LabDiet 5010, LabDiet, Richmond, IN, USA) and purified water (in 500 mL drinking water bottles). Visual health inspection was performed twice daily, and cleaning the cages was performed once a week.

### 2.2. Treatments

This was an experimental prospective–comparative study. All measurements were taken by a single trained and unblinded evaluator. Before starting the experimental phase, rats underwent a habituation period of 15 days to the evaluator’s presence and animal handling. The animals were randomly divided into six treatments by a random number generator (Microsoft Excel; Microsoft 365). Ten rats were assigned to each treatment (five males and five females), according to the euthanasia method:

Pentobarbital: pentobarbital (pentobarbital, Aranda^®^, Querétaro, QRO, Mexico) overdose at 400 mg/kg IP with a 3 mL sterile syringe (Ambiderm^®^, Zapopan, JAL, México) [31]. The dose was calculated through a pilot study (no published data), using the minimal and maximal doses that appear in Reimer et al.’s [32] study. CO_2_: CO_2_ overdose inside an acrylic euthanasia chamber (Acrifactory, Mexico) (32.5 × 42 × 21 cm). The flow rate was set to 30% of the chamber volume/min [33]. Decapitation: decapitation using a rodent guillotine (51330, Senna, Ciudad de México, CDMX, Mexico) [31]. Iso: inhalation of isoflurane (Fluriso, VET ONE^®^, Boise, ID, India) using the open-drop exposure method (two cotton swabs soaked with 2 mL of isoflurane each). The dose was calculated using Risling et al. [34] and de Brito’s [35] study as a basis. The cotton swabs were placed where animals could not have direct contact with the inhalant anesthetic drug. K + X: ketamine (Ketamin-Pet, Aranda^®^, Querétaro, QRO, Mexico) + xylazine (Procin, Pisa Agropecuaria^®^, Guadalajara, JAL, Mexico) overdose, at doses of 450 mg/kg IP and 45 mg/kg IP, respectively [33]. K + CO_2_: the combination of ketamine (100 mg/kg IP) + CO_2_ (after 5–10 min of ketamine administration) [36]. No control animals were included in the experimental design since the aim of the study was to compare the different injectable, inhalant, and physical euthanasia methods.

### 2.3. Evaluation Events

Four events were recorded. Basal: video recording for five minutes, 24 h before the euthanasia method, inside the housing room; Ti_1_: three minutes of recording before the application of the euthanasia in the test room. On the trial day, the rats were moved from the housing room to the test room (average temperature of 22.9 ± 0.5 °C and 52% humidity), allowing rest and room acclimatization for 30 min before starting the trial; Ti_2_: during the application of the method (e.g., while the animal received the IP dose of pentobarbital, ketamine + xylazine, or while it was inside the induction chamber or placed in the guillotine); Ti_3_: immediately after the application of the euthanasia method until loss of the righting reflex (LORR) as a sign of unconsciousness. The absence of palpebral, interdigital, and righting reflexes was also used to confirm the euthanasia method. Cessation of breathing and heart rate was also used to confirm the death of the rats.

### 2.4. Rat Grimace Scale Recording and Scoring

To obtain the RGS score, continuous recording with two high-resolution (1920 × 1080) digital cameras (Ixy 650, Cannon^®^, Ohta-ku, NRT, Japan) was performed during each evaluation event. The cameras were placed on both sides of the animal (front and side) to capture headshots of the rats before, during, and after each euthanasia treatment. Cameras were mounted on tripods at a distance of approximately 20 cm from the rats.

**Figure 1:**
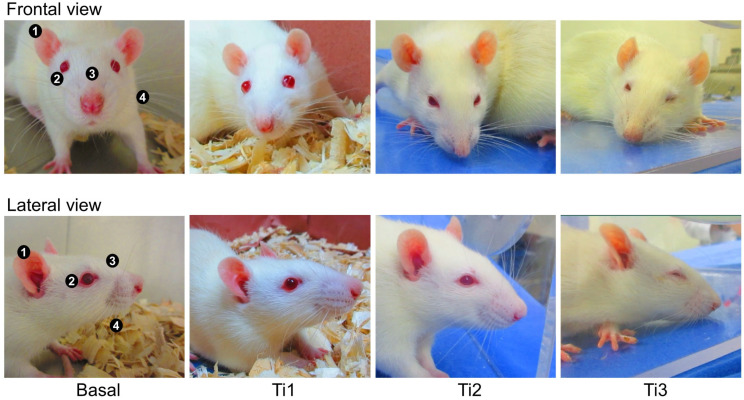
Example of the FAU evaluated in the sixty rats and the assigned score. (1) ear change; (2) orbital tightening; (3) nose/cheek flattening; and (4) whisker change.

The videos were saved as MP4 format files to be analyzed by a coder in video editing software (Adobe Premiere Pro, Adobe, San José, CA, USA). By watching the videos at a speed of 0.5×, 10 still images of the rats’ faces were taken at each evaluation event (Basal, Ti_1_, Ti_2_, and Ti_3_) [37], obtaining 40 images per rat when a clear front or lateral view of the head was observed. Scoring of the images was performed by a different single blinded evaluator according to Sotocinal’s [22] study using four FAUs: orbital tightening, nose/cheek flattening, ear change, and whisker change (Figure 1). Using a score scale from 0 to 2, where 0 = not present, 1 = moderately present; and 2 = obviously present, the maximum score obtainable was 8. To obtain the final score, the 10 images per evaluation event in each FAU were summed to obtain a mean value. For each rat, the mean values of the four FAUs were summed, and a mean total score was used in the analysis.

### 2.5. Procedures

During Basal, recording for RGS was performed for five minutes inside the housing room. The cameras were placed on tripods on two sides of the polycarbonate cages. Twenty-four hours later, the rats from the corresponding treatment were moved from the housing room to a testing room to perform euthanasia away from the rest of the housed animals. A period of 30 min was established so the rats could acclimatize to the testing room and avoid transport stress. During this time, the cameras were mounted on tripods in the testing room, in different locations according to the treatment (e.g., if it was an inhalant method, the cameras were placed on both sides of the euthanasia chamber). After the preparation of the room and acclimatization of the animals and before the application of the euthanasia method, three-minute recordings were performed. After this and during Ti_2_ and Ti_3_, a continuous recording was taken until LORR; therefore, the length of the video in these events differed according to the treatment.

### 2.6. Statistical Analyzes

The sample size was calculated using G*Power 3.1.9.7 (Heinrich-Heine-Universität Düsseldorf, Düsseldorf, Germany). The sample size considered an α error probability of 0.05, confidence level of 95%, 1-β error probability of 0.90, and correction among repeated measures of 0.5 for the six treatments at five events. The GraphPad Prism 10.0.2 (San Diego, CA, USA) statistical package was used to analyze the data. The Kolmogorov–Smirnov test was performed to establish data normality in the data set collected from the FAUs. Descriptive statistics were obtained, expressing the results as mean ± standard deviation (SD). A linear mixed model for repeated measures was used to evaluate the effect of the six treatments (Pentobarbital, CO_2_, Decapitation, Iso, K + X, and K + CO_2_), in the four events (basal, Ti_1_, Ti_2_, and Ti_3_,) for the total RGS scores. Multiple comparisons of means were performed with the post-hoc Tukey test. Correlations between the four FAUs and the treatments were calculated using Spearman correlation coefficients. All values with *p* < 0.05 were considered statistically significant.

### 2.7. Ethical Statement

The present study was approved by the Committee for the Care and Use of Laboratory Animals (INRLGII/CICUAL/014/2021) of National Institute of Rehabilitation Luis Guillermo Ibarra-Ibarra.

The handling and care of the laboratory animals was in accordance with the Mexican Norm NOM-062-ZOO-1999 for Laboratory Animals [38]. All dead animals were disposed by incineration, following the NOM-062-ZOO-1999 [39].

## 3. Results

A total of 758.58 min of recording and 2400 images taken from the videos were evaluated. Table 1 shows the total RGS score (expressed as mean ± SD) of the six euthanasia methods during the four events. When making comparisons between treatments, no significant differences were found at Ti_2_ (*p* < 0.0001) in rats undergoing euthanasia by decapitation and a combination of ketamine + xylazine, obtaining scores of 0.6 ± 0.26 and 0.6 ± 0.16, respectively. In contrast, at Ti_3_, significant differences (*p* < 0.0001) were recorded for CO_2_ and isoflurane, obtaining the highest scores of 0.9 ± 0.18 and 1.2 ± 0.20, respectively. The analysis between events shows an expected significant increase in the RGS score in all treatments, from Basal/Ti_1_ to Ti_2_ and Ti_3_ (*p* < 0.05). However, the largest increase in scores was found for Decapitation during Ti_2_ (*p* = 0.0052) and for Iso during Ti_3_ (*p* < 0.0001).

Correlations between treatments according to the FAU were obtained. Table 2 shows the correlation coefficients for “ear change”, where a statistically significant (*p* < 0.0001) moderate correlation (r = 0.685) was found between rats receiving CO_2_ and isoflurane.

Similar to “ear change”, “orbital tightening” had significant moderate correlations between CO_2_ and Iso (r = 0.540) (*p* < 0.0001), while Iso rats had a negative moderate correlation with rats receiving ketamine + xylazine (r = −0.465) (*p* < 0.0001). A statistically significant correlation was also found between Decapitation and K + X treatments (r = 0.466) (*p* < 0.0001) (Table 3).

Regarding “nose/cheek flattening”, statistically significant (*p* < 0.0001) moderate correlations (r = 0.670) were found between CO_2_ and Iso (Table 4), a similar case as the one observed in Table 5 for “whisker change”, where CO_2_ and Iso had a moderate correlation (r = 610) (*p* < 0.0001). In the same FAU, K + CO_2_ was moderately correlated with both CO_2_ (r = 577) (*p* < 0.0001) and Iso (r = 0.493) (*p* <0.0001).

## 4. Discussion

Regardless of the euthanasia method, the handling, and procedure, per se (e.g., IP injection), might imply stress, distress, and pain for laboratory rodents. This was observed in the present study in all euthanasia methods as an expected increase in the RGS score from Basal/Ti_1_ to Ti_2_ and Ti_3_. At Ti_2_, the highest scores were reported for Decapitation and K + X, followed by K + CO_2_, while Iso and CO_2_ registered the highest values at Ti_3_. It is known that an average score of 0.67 in the RGS is considered the intervention threshold to administer analgesic drugs during a painful event [40,41]. Therefore, the facial changes observed during and after the application of the euthanasia methods could be related to the nociceptive pathway and its motor responses.

In rats, pain can elicit changes in facial expression due to routine practices, handling techniques, surgical procedures, and analgesic protocols [25,27,28]. Nonetheless, to discuss the main findings of the present study it is essential to acknowledge that discriminating pain from the potential stress/distress that animals might perceive during euthanasia is challenging. This was reported in a recently published paper that constitutes the first study phase of the present research, where infrared thermography was used to evaluate the level of stress/distress of rats undergoing the same euthanasia methods [30]. As the conscious perception of acute pain and the nociceptive pathway is triggered by a real or potential noxious stimulus, this represents a temporary threat to homeostasis and, thus, stress [42,43]. Moreover, both stress/distress and pain are overlapping processes that share neuronal pathways and can activate similar brain structures that are interconnected [44]. Furthermore, negative mental states such as fear and anxiety—not assessed in the present study—might also influence pain perception and the consequent facial expression of animals by sensitizing peripheral nociceptors as the so-called stress-induced hyperalgesia [43,45,46]. Therefore, when interpreting the RGS during euthanasia, one must acknowledge that this response could be the result of a series of negative states, and regarding it only as pain would be inappropriate.

Considering that all treatments increased their RGS scores from Basal/Ti_1_ to Ti_2_ and Ti_3_, some studies have reported the RGS scores related to the effect of the euthanasia method. For example, Khoo et al. [27] determined that a combination of sodium pentobarbital and lidocaine for euthanasia reduced the frequency of writhing but did not influence the RGS, where low values were registered between 0.44 ± 0.14 and 0.37 ± 0.11. The obtained scores by Khoo et al. [27] were higher than the ones reported in the present study (0.1 ± 0.07 at Ti_2_ and 0.2 ± 0.14 at Ti_3_). However, both findings suggest that IP administration of pentobarbital might cause mild pain—together with potential stress and discomfort—as reported by Svendsen et al. [47] in rats, where an increase in c-fos-expressing neurons in the spinal cord is related to nociception. In contrast, Reimer et al. [32] compared pentobarbital, saline, and vehicle control groups in Sprague Dawley and Wistar rats to determine the level of pain in both strains. It was found that RGS scores were above 0.67 only in the saline and vehicle groups, which were attributed to a higher frequency of writhing and back arching. This might suggest that IP injection and the restraining method could also be involved in the behavioral and physiological response of animals.

As mentioned by Laferriere and Pang [48], although pentobarbital is a killing method recommended over inhalational agents (e.g., CO_2_ and isoflurane), pentobarbital is a highly alkaline solution with a pH between 11 and 12. Hence, IP injection might cause pain and irritation of the peritoneal cavity and viscera because the tissular nonirritating pH is approximately 4.5–8.0 [9,49]. Pain-related behaviors reported after pentobarbital administration are writhing, vocalization, increase in locomotion, or flinching [48], and the incidence of said behaviors can range between 36 and 46% with low (200 mg/kg) and high doses (800 mg/kg), respectively. Nonetheless, no changes or no statistical differences have been reported in mice receiving IP pentobarbital, implying that barbiturates might cause peritoneal inflammation, but fast action of the euthanasia method does not elicit signs of stress or pain [10]. Likewise, Boivin et al. [50] determined that IP administration of pentobarbital-phenytoin caused less stress to mice, according to the ACTH concentrations, than inhalant agents (CO_2_, and isoflurane anesthesia followed by CO_2_ inhalation), as could also be assumed in the present research, where Pentobarbital rats had the lowest RGS scores during and after the administration of pentobarbital.

A similar case is observed in K + X. At Ti_2_, the K + X rats’ RGS score was 0.6 ± 0.16, one of the two highest values during this event (together with Decapitation). These findings might be due to potential pain from IP injection or to ketamine’s chemical properties. It is known that ketamine has a low pH (3.5 to 4.1) to facilitate solubilization [51]. However, after parenteral administration, ketamine has been associated with tissular irritation, pain, and muscle damage at the injection site [52], and repeated IP anesthesia with ketamine + xylazine in mice increased the MGS score (approximately between 0.60 and 1.20) and caused short-term anxiety-like behaviors [53]. Anesthesia using the same combination caused more severe muscle and tissue necrosis in Wistar Han rats than those receiving ketamine + dexmedetomidine. Although histopathologic changes were not assessed in the present study, the high scores observed in Ti_2_ in both groups receiving ketamine IP (K + X and K + CO_2_) could be associated with ketamine’s properties.

Regarding the differences between both injectable methods, the properties of each drug might be related to the obtained RGS scores. On the one hand, pentobarbital enhances the GABAergic system and has an anxiolytic and muscle relaxant effect [54]. While the K + X combination acts on dopaminergic and histaminergic fibers, with a dissociative state that affects the ascending reticular system [55], which might explain the increased score in this group. Additionally, it is known that handling techniques can also trigger a stress response [56]. This might influence facial expressions. Therefore, the increased RGS scores observed in K + X animals, when compared to inhalational agents, might be due to the highest stress prompted by the restrain technique to perform IP injection and further research would be required to discriminate this effect.

During Ti_2_, the other treatment that recorded the highest RGS score was decapitation (0.6 ± 0.26). Decapitation is a highly controversial method, not only because it might be aesthetically unpleasant but also because of the potential pain that animals might perceive [11,57]. Decapitation of small rodents is an acceptable method because several authors have reported a short period of consciousness after head detachment from the spinal cord (between 3 and 15 s) [14], which would limit the degree of stress or pain that animals could perceive. Through electroencephalography (EEG), Gagea-Iurascu and Craig [12] and Mikesha and Klemm [58] mention that cerebral activity is present between 2.7 and 40 s after decapitation, while Cartner et al. [59] recorded a decrease in cortical function and visual evoked potentials of mice within 15–20 s and 10–15 s, respectively. These results show that decapitation leads to a rapid dysfunction of cerebral activity. Moreover, the immediate loss of blood flow to the brain hastens hypoxia, anoxia, and subsequent death [60]. Nonetheless, other studies have associated decapitation with nociception due to the presence of low voltage and fast EEG activity—a sign of conscious perception of pain [13]. Likewise, an increase in median frequency (F50) and spectral edge frequency (F95) within the first 15 s after decapitation have been reported [15], an EEG change that indicates nociception in rats [61].

The possible short-term presence of stress, nociception, conscious pain awareness, and the high RGS scores obtained in the present research would need further study combining behavioral, endocrine, physiological, and EEG assessment. However, a possible explanation for why nociception/pain might be present could be due to the activation of peripheral nociceptors located in the skin and muscles of the cervical region of rodents and to the potential sensitization of nociceptors due to negative states derived from decapitation (e.g., fear). Although the loss of consciousness is fast after decapitation, before head detachment, tissular damage to said structures would trigger the nociceptive pathway (e.g., transduction, transmission, modulation, projection, and perception) culminating in pain perception for a couple of seconds before the cessation of nervous signaling [7,21,62]. It is known that after decapitation there is an anatomical disconnection between the mesencephalon and the cardiorespiratory centers—leading to death—and that the electric signaling present post-decapitation cannot solely be attributed to nociception since it has been found in healthy anesthetized animals or during REM sleep [14,33]. Thus, while animals might not be able to perceive pain within 3 s after decapitation, making it a humane killing method, there could be a debate about the degree of the short-term pain or nociceptive activation that laboratory rodents might perceive due to tissular damage before reaching death, requiring future comprehensive studies about this topic.

Regarding the inhalational methods (CO_2_ and Iso), an interesting result was observed during Ti_3_. At Ti_2_, both methods obtained a score of 0.2 and 0.3, respectively, being lower than Decapitation, K + X, and K + CO_2_. In contrast, at Ti_3_, the highest RGS scores were found in CO_2_ (0.9 ± 0.18) and Iso (1.2 ± 0.20). In this sense, regarding CO_2_, there are no studies where RGS was used during the euthanasia or anesthesia of rats; however, some studies in rodents have found that animals perform active and passive defense behaviors when exposed to CO_2_ and that physiological responses, such as bradycardia and corticosterone increases, are present [63]. Similarly, Marquardt et al. [2] concluded that glucose, adrenaline, and noradrenaline increased in mice exposed to high concentrations of CO_2_ (60% and 100%), in contrast to isoflurane and sevoflurane inhalation. When assessing cortical EEG changes due to CO_2_ euthanasia, Thurauf et al. [64] reported that different concentrations of CO_2_ evoked a negative mucosal potential due to painful stimulation. Apart from pain responses, CO_2_ is aversive to rodents, causing behavioral alterations, such as escape behaviors, vocalizations, freezing, and the activation of fear/anxiety brain regions (e.g., amygdala, dorsomedial region of the hypothalamus, hypothalamus, and bed nucleus of the stria terminalis) [63].

The suggestion that CO_2_ exposure elicits pain in rodents has been translated from studies in humans, where concentrations between 50 and 100% were regarded as highly unpleasant, stressful, and painful [57,65]. The possible association between pain and CO_2_ has been related to carbonic anhydrase and the formation of carbonic acid within mucosal surfaces when CO_2_ interacts with water [16,66]. Leach et al. [67] mention that rodents exposed to CO_2_ increase stress-induced grooming as a potential indicator of mucosa irritation, particularly for high concentrations and pre-filled chambers. Furthermore, CO_2_ inhalation decreases the pH (<7.2), resulting in tissue acidosis and the activation of nociceptors, such as ASIC [16], or polymodal-nociceptive neurons—also known as wide dynamic range nociceptive neurons—with CO_2_ concentrations up to 40% [68,69].

On the other hand, isoflurane euthanasia significantly increased the RGS score to 1.2 ± 0.20 during Ti_3_, recording the highest score in all treatments during all events. Isoflurane is known to be a moderate pungent agent, more than halothane and enflurane, that causes high aversion to rodents [9,17]. There are no studies assessing isoflurane and RGS during euthanasia; nonetheless, the present findings regarding isoflurane agree with the Miller et al. [70,71] studies in rats and mice, where the authors reported the influence of the anesthetic on the RGS. The authors found that isoflurane increased RGS scores after 12 min of anesthesia [70], while in DBA/2 mice, the MGS scores increased in animals receiving the halogenated drug [71], results that might be associated with the residual effects of isoflurane and its pharmacological properties. Likewise, single and repeated exposures to isoflurane anesthesia in female mice resulted in higher RGS scores in the first 30 min after anesthesia (approximately 0.8 and 1.2) and reduced burrowing behavior and food intake [72]. Furthermore, Wong et al. [73] determined that CO_2_ is more aversive than isoflurane, and that isoflurane sedation before CO_2_ euthanasia is a refinement. Even recent studies have proposed the isoflurane “drop method” to anesthetize rodents before CO_2_ euthanasia in mice [74].

Considering the available literature regarding isoflurane anesthesia and the application of the RGS, additional studies are necessary to determine if, during euthanasia, the high RGS score found in Iso rats could be completely associated with stress, pain, or might be a pharmacological effect of isoflurane. In this sense, isoflurane inhibits acetylcholine receptors, causing dose-dependent muscle relaxation of both skeletal and smooth muscles [75]. This property could influence the innervation of mimetic muscles used to evaluate facial expressions in rodents, as presented in this research. A period of 30 min post laparotomy or 1 h after surgical anesthesia with isoflurane has been used by authors, such as Klune et al. [26] and Leung et al. [25], to evaluate the RGS without the confounding factor of isoflurane residual effects.

The findings regarding both inhalational agents can also be observed in the correlations presented in Table 2, Table 3, Table 4 and Table 5 and the potential noxious and pharmacological effects of CO_2_ and isoflurane. For all FAUs, CO_2_ and Iso were moderately and positively correlated. This suggests that inhalational methods for euthanasia cause a similar facial response (e.g., both methods cause a progressive increase in the “eye tightening” and “ear position”). While this might be associated with the activation of nociceptors and pungency level, the pharmacological effect of the drugs might be also altering the RGS score. Likewise, the negative moderate correlation found between Iso and K + X could explain the effect that the drug can have on the facial response of animals. In this case, Iso animals differed from K + X treatment possibly due to the ketamine-induced catalepsy that maintains open eyes [76], while isoflurane, due to its action on the GABAergic fibers, has the opposite effect by closing the eyes and, therefore, increasing the RGS.

Refinement of current euthanasia methods for laboratory rodents requires recognizing that distress, nociception, and pain might be present during the process. This also helps to advocate for alternatives, such as the combination of local anesthetics (e.g., lidocaine, bupivacaine) with IP drugs, resulting in a lower frequency of abdominal writhing and a lower incidence of ultrasonic vocalizations related to pain [27]. In the present study, a combination of ketamine + CO_2_ increased the RGS during Ti_2_ (possibly due to the IP injection) but only by an average of 0.2, while in Ti_3_, the RGS score decreased. In contrast, Valentine et al. [77] reported that rodents receiving CO_2_ without premedication showed fewer signs of stress and pain (e.g., lower cerebral expression of c-fos) than its combination with acepromazine, midazolam, or isoflurane. A reason why ketamine in combination with CO_2_ did not have as high an RGS score as CO_2_ used alone might be due to the inhibitory properties of ketamine in NMDA receptors, inducing amnesia, analgesia, and prevention of central sensitization [9]. Ketamine’s mechanism of action prevents neural signaling from second-order neurons to supraspinal structures, decreasing thalamocortical, limbic, and reticular activity (regions necessary for conscious pain recognition) [78,79].

### Limitations and Recommendations for Future Research

Nociception and pain have a multidimensional nature with sensory/discriminative, affective/motivational, and cognitive components [80]. Due to this, nociception and pain assessment and recognition in animals require the comprehensive study of several parameters that could help to evaluate the different dimensions of pain. In this sense, the main limitation of the present study is the lack of additional methods to assess pain or stress. For example, it would have been useful to use behavior-based scales (e.g., composite behavioral scale) to correlate pain- or stress-related behaviors to the RGS scores. Moreover, technologies (e.g., EEG or electrophysiological techniques to measure nociceptor activity) or comparison with pain-related biomarkers in rodents, such as corticosterone, adrenaline, noradrenaline, c-fos, or ultrasonic vocalizations, would have helped to bring a comprehensive evaluation. When using infrared thermography as a non-invasive technique to evaluate the stress/distress caused during the euthanasia of Wistar rats, a significant effect of inhalational agents on the thermal response of animals was found, which might be associated with higher levels of stress/distress [30].

Additionally, the lack of a control group to compare the euthanasia methods would have helped to clarify the impact of fixation and injection itself, as shown in a study where the IP administration of a non-irritant agent increased RGS scores [32]. Using a control group to compare the administration of injectable agents might be necessary to objectively determine the sensitivity and robustness of the RGS when applied to the euthanasia period to assess pain [81]. Therefore, an overall evaluation of pain, considering control animals, is necessary, particularly in research animals.

In the present study, it was shown that the pharmacological effect of isoflurane might have influenced the RGS. However, this effect is not limited to isoflurane but might also be present in other euthanasia methods where anesthetics are used (e.g., pentobarbital, ketamine + xylazine). As some anesthetics cause sedation and muscle relaxation [82], RGS scores in these animals might increase or decrease independently of the degree of stress/pain. This is relevant because drug administration might negatively influence facial expression. Hence, research on facial expressions needs to include other parameters to prevent bias or misinterpretations.

Also, the time when a change in facial expression can be evaluated in rats during euthanasia might need to consider the time before unconsciousness or LORR. For example, the time to LORR in the present animals has been already published in Domínguez-Oliva et al.’s [30] study. The range for the time to LORR (in seconds) was between 6.2 ± 4.0 s and 122.7 ± 21.8 s for Decapitation and K + CO_2_, respectively. As the time to LORR highly differs among the different euthanasia methods due to the pharmacological properties of the drug or the technique, the application of the RGS to evaluate the facial changes in animals must consider the possible effect of time. For example, although facial micro-expressions (generated in almost 500 ms in humans) have been reported in horses [83], laboratory rodents are prey animals known to covet stress/distress or pain as a survival strategy [18]. Thus, apart from recognizing that facial expression and its scoring are highly influenced by other negative states, such as stress and fear, future research could aim to standardize the amount of time needed to evaluate facial muscle changes.

According to the obtained results, it is suggested that during the application of the euthanasia method, RGS scores might increase due to tissular damage and potential activation of peripheral nociceptors, eliciting short-term pain when using injectable and physical methods. Some authors have proposed oral administration for premedication before the actual euthanasia method to refine euthanasia. For example, Rodríguez-Sánchez et al. [84] recommended the use of oral tiletamine + zolazepam before euthanasia to induce sedation and decrease aversion. Nonetheless, Dudley and Boivin [85] found in mice that orally ingested pentobarbital solution in cookie dough did not induce LORR, unconsciousness, or death. Hence, evaluating through the RGS, and other pain-related markers, the application of novel strategies with current euthanasia methods could help to perform a complete and objective evaluation. Nonetheless, the effects that anesthetics (e.g., pentobarbital and isoflurane) might have on facial expressions and mimetic muscle control, which could limit the behavioral assessment of euthanasic drugs, must be acknowledged [32,70].

In the present study, only Wistar rats were assessed. However, some reports have shown differences in behavioral responses associated with pain between different strains of rats (e.g., Sprague Dawley and Wistar) [32]. Winter et al. [86] found significant differences between Sprague Dawley, Wistar, Long Evans, and Wistar-Kyoto rats exposed to CO_2_. Freezing was prevalent in Long Evans and Wistar/Kyoto rats, and behavioral responses, such as rearing and grooming, also depended on the strain, as well as the expression of serotonergic, noradrenergic, and dopaminergic neurons. Thus, these results show that nociception and pain evaluation require consideration of the strain and the possible implications when selecting the euthanasia method, not only from a physiological and endocrine perspective but as a thorough assessment.

Lastly, it is important to mention that finding the appropriate instrument for recording stress, suffering, or pain in animals remains challenging for researchers. The present article is an approximation of the question and scientists should strive for improvements in the sense of refinement, particularly during the euthanasia methods for laboratory animals.

## 5. Conclusions

According to the high RGS scores observed during the application of injectable (pentobarbital, ketamine + xylazine) and physical methods (decapitation), the findings of the present study suggest that Wistar rats can perceive short-term nociception when compared to inhalational agents. In contrast, during the first minutes after performing euthanasia, inhalant agents record the highest RGS scores, particularly isoflurane. While this could be associated with the activation of peripheral nociceptors, pungency, or mucosal irritation—variables that were not assessed in the present research—the pharmacological effect of isoflurane and other anesthetics as muscle relaxants need to be considered to objectively interpret changes in the facial expression of rats.

Therefore, the RGS could be applied as a method to evaluate and reconsider some of the euthanasia methods. However, as pain is multidimensional, the behavioral, physiological, and electrophysiological assessments must be used together with facial expression ratings to measure the level of pain that current euthanasia procedures evoke in research animal models.

## Figures and Tables

**Table 1:** RGS scores (mean ± standard deviation) of the six euthanasia methods during the four events.

Treatments	Basal	Ti_1_	Ti_2_	Ti_3_	*p*-Value
Pentobarbital	0.0 ± 0.0 ^a,1^	0.0 ± 0.0 ^a,1^	0.1 ± 0.07 ^a,b,1^	0.2 ± 0.14 ^b,1^	***p* = 0.0294**
CO_2_	0.0 ± 0.0 ^a,1^	0.0 ± 0.0 ^a,1^	0.2 ± 0.11 ^b,1,2^	0.9 ± 0.18 ^c,2^	***p* < 0.0001**
Decapitation	0.0 ± 0.0 ^a,1^	0.0 ± 0.0 ^a,1^	0.6 ± 0.26 ^b,3^	0.3 ± 0.24 ^a,b,1^	***p* = 0.0052**
Iso	0.0 ± 0.0 ^a,1^	0.0 ± 0.0 ^a,1^	0.3 ± 0.16 ^b,1,2,3^	1.2 ± 0.20 ^c,2^	***p* < 0.0001**
K + X	0.0 ± 0.0 ^a,1^	0.0 ± 0.0 ^a,1^	0.6 ± 0.16 ^b,3^	0.3 ± 0.12 ^c,1^	***p* < 0.0001**
K + CO_2_	0.0 ± 0.0 ^a,1^	0.0 ±0.0 ^a,1^	0.4 ± 0.15 ^b,2,3^	0.5 ± 0.10 ^b,1^	***p* < 0.0001**
*p*-value	*p* > 0.05	*p* > 0.05	***p* < 0.0001**	***p* < 0.0001**	

^a,b,c^ Different letters represent statistically significant differences between events. ^1,2,3^ Different numbers represent statistically significant differences between treatments. Basal: 24 h before the procedure; Ti_1_: three minutes before the procedure; Ti_2_: during the application of the euthanasia method; Ti_3_: immediately after the application until LORR.

**Table 2:** Correlation matrix for the FAU “Ear change”.

	Pentobarbital	CO_2_	Decapitation	Iso	K + X	K + CO_2_
Pentobarbital	1					
CO_2_	0.354 *p* < 0.001	1				
Decapitation	−0.070 *p* = 0.326	−0.086 *p* = 0.226	1			
Iso	0.391 *p* < 0.001	0.685 *p* < 0.001	0.001 *p* = 0.990	1		
K + X	0.155 *p* = 0.028	−0.053 *p* = 0.456	0.151 *p* = 0.033	−0.079 *p* = 0.269	1	
K + CO_2_	0.345 *p* < 0.001	0.259 *p* < 0.001	0.172 *p* = 0.015	0.295 *p* < 0.001	0.450 *p* < 0.001	1

**Table 3:** Correlation matrix for the FAU “Orbital tightening”.

	Pentobarbital	CO_2_	Decapitation	Iso	K + X	K + CO_2_
Pentobarbital	1					
CO_2_	0.236 *p* < 0.001	1				
Decapitation	−0.038 *p* = 0.593	−0.275 *p* < 0.001	1			
Iso	0.239 *p* < 0.001	0.540 *p* < 0.001	0.140 *p* = 0.049	1		
K + X	0.141 *p* = 0.047	−0.343 *p* < 0.001	0.395 *p* < 0.001	−0.465 *p* < 0.001	1	
K + CO_2_	0.070 *p* = 0.322	−0.254 *p* < 0.001	0.466 *p* < 0.001	−0.389 *p* < 0.001	0.622 *p* < 0.001	1

**Table 4:** Correlation matrix for the FAU “Nose/cheek flattening”.

	Pentobarbital	CO_2_	Decapitation	Iso	K + X	K + CO_2_
Pentobarbital	1					
CO_2_	0.164 *p* = 0.020	1				
Decapitation	−0.034 *p* = 0.633	−0.118 *p* = 0.097	1			
Iso	0.266 *p* < 0.001	0.670 *p* < 0.001	−0.154 *p* = 0.029	1		
K + X	0.041 *p* = 0.568	0.177 *p* = 0.012	0.087 *p* = 0.221	0.107 *p* = 0.13	1	
K + CO_2_	0.339 *p* < 0.001	0.387 *p* < 0.001	−0.250 *p* < 0.001	0.441 *p* < 0.001	0.242 *p* = 0.001	1

**Table 5:** Correlation matrix for the FAT “Whisker change”.

	Pentobarbital	CO_2_	Decapitation	Iso	K + X	K + CO_2_
Pentobarbital	1					
CO_2_	0.369 *p* < 0.001	1				
Decapitation	−0.221 *p* = 0.001	−0.341 *p* < 0.001	1			
Iso	0.206 *p* = 0.003	0.610 *p* < 0.001	−0.014 *p* = 0.840	1		
K + X	0.163 *p* = 0.021	0.180 *p* = 0.011	0.102 *p* = 0.149	0.252 *p* < 0.001	1	
K + CO_2_	0.415 *p* < 0.001	0.577 *p* < 0.001	−0.212 *p* = 0.003	0.493 *p* < 0.001	0.398 *p* < 0.001	1

## Data Availability

The data presented in this study are contained within the article.

## References

[1] Prescott M.J., Lidster K. (2017). Improving quality of science through better animal welfare: The NC3Rs strategy. Lab. Anim..

[2] Marquardt N., Feja M., Hünigen H., Plendl J., Menken L., Fink H., Bert B. (2018). Euthanasia of laboratory mice: Are isoflurane and sevoflurane real alternatives to carbon dioxide?. PLoS ONE.

[3] Kroll T., Kornadt-Beck N., Oskamp A., Elmenhorst D., Touma C., Palme R., Bauer A. (2021). Additional Assessment of Fecal Corticosterone Metabolites Improves Visual Rating in the Evaluation of Stress Responses of Laboratory Rats. Animals.

[4] Baumans V. (2004). Use of animals in experimental research: An ethical dilemma?. Gene Ther..

[5] Hirose M., Rajendram R., Patel V.B., Preedy V.R., Martin C.R. (2022). Nociception during surgery. Features and Assessments of Pain, Anaesthesia, and Analgesia.

[6] Raja S.N., Carr D.B., Cohen M., Finnerup N.B., Flor H., Gibson S., Keefe F.J., Mogil J.S., Ringkamp M., Sluka K.A. (2020). The revised International Association for the Study of Pain definition of pain: Concepts, challenges, and compromises. Pain.

[7] Hernández-Avalos I., Flores-Gasca E., Mota-Rojas D., Casas-Alvarado A., Miranda-Cortés A.E., Domínguez-Oliva A. (2021). Neurobiology of anesthetic-surgical stress and induced behavioral changes in dogs and cats: A review. Vet. World.

[8] Descovich K. (2017). Facial expression: An under-utilised tool for the assessment of welfare in mammals. ALTEX.

[9] Flecknell P. (2016). Laboratory Animal Anaesthesia.

[10] Dutton J.W., Artwohl J.E., Huang X., Fortman J.D. (2019). Assessment of Pain Associated with the Injection of Sodium Pentobarbital in Laboratory Mice (*Mus musculus*). J. Am. Assoc. Lab. Anim. Sci..

[11] Turner M.D. (2023). “The Most Gentle of Lethal Methods”: The Question of Retained Consciousness Following Decapitation. Cureus.

[12] Gagea-Iurascu M., Craig S., Suckow M.A., Stevens K.A., Wilson R.P. (2012). Euthanasia and Necropsy. The Laboratory Rabbit, Guinea Pig, Hamster, and Other Rodents.

[13] Derr R. (1991). Pain perception in decapitated rat brain. Life Sci..

[14] Holson R.R. (1992). Euthanasia by decapitation: Evidence that this technique produces prompt, painless unconsciousness in laboratory rodents. Neurotoxicol. Teratol..

[15] Kongara K., McIlhone A., Kells N., Johnson C. (2014). Electroencephalographic evaluation of decapitation of the anaesthetized rat. Lab. Anim..

[16] Boivin G.P., Hickman D.L., Creamer-Hente M.A., Pritchett-Corning K.R., Bratcher N.A. (2017). Review of CO₂ as a Euthanasia Agent for Laboratory Rats and Mice. J. Am. Assoc. Lab. Anim. Sci..

[17] De la Torre C., De la Torre C., Jiménez P., Ramos M.M., De la Torre G. (2012). Anestesia para colonoscopia: Anestesia inhalatoria con sevoflurano frente a anestesia intravenosa con propofol. Sanid. Mil..

[18] Whittaker A.L., Howartha G., Howarth G.S. (2014). Use of spontaneous behaviour measures to assess pain in laboratory rats and mice: How are we progressing?. Appl. Anim. Behav. Sci..

[19] Darwin C. (1872). The Expressions of the Emotions in Man and Animals.

[20] Deuis J.R., Dvorakova L.S., Vetter I. (2017). Methods Used to Evaluate Pain Behaviors in Rodents. Front. Mol. Neurosci..

[21] Domínguez-Oliva A., Mota-Rojas D., Hernández-Avalos I., Mora-Medina P., Olmos-Hernández A., Verduzco-Mendoza A., Casas-Alvarado A., Whittaker A.L. (2022). The neurobiology of pain and facial movements in rodents: Clinical applications and current research. Front. Vet. Sci..

[22] Sotocinal S.G., Sorge R.E., Zaloum A., Tuttle A.H., Martin L.J., Wieskopf J.S., Mapplebeck J.C., Wei P., Zhan S., Zhang S. (2011). The Rat Grimace Scale: A Partially Automated Method for Quantifying Pain in the Laboratory Rat via Facial Expressions. Mol. Pain.

[23] Whittaker A.L., Liu Y., Barker T.H. (2021). Methods Used and Application of the Mouse Grimace Scale in Biomedical Research 10 Years on: A Scoping Review. Animals.

[24] Philips B.H., Weisshaar C.L., Winkelstein B.A. (2017). Use of the Rat Grimace Scale to evaluate neuropathic pain in a model of cervical radiculopathy. Comp. Med..

[25] Leung V., Zhang E., Pang D.S. (2016). Real-time application of the Rat Grimace Scale as a welfare refinement in laboratory rats. Sci. Rep..

[26] Klune C.B., Larkin A.E., Leung V.S.Y., Pang D. (2019). Comparing the Rat Grimace Scale and a composite behaviour score in rats. PLoS ONE.

[27] Khoo S.Y.-S., Lay B.P.P., Joya J., McNally G.P. (2018). Local anaesthetic refinement of pentobarbital euthanasia reduces abdominal writhing without affecting immunohistochemical endpoints in rats. Lab. Anim..

[28] Akintola T., Raver C., Studlack P., Uddin O., Masri R., Keller A. (2017). The grimace scale reliably assesses chronic pain in a rodent model of trigeminal neuropathic pain. Neurobiol. Pain.

[29] Kovalcsik R., Devlin T., Loux S., Martinek M., May J., Pickering T., Tapp R., Wilson S., Serota D. (2006). Animal reuse: Balancing scientific integrity and animal welfare. Lab Anim..

[30] Domínguez-Oliva A., Hernández-Ávalos I., Olmos-Hernández A., Villegas-Juache J., Verduzco-Mendoza A., Mota-Rojas D. (2023). Thermal response of laboratory rats (*Rattus norvegicus*) during the application of six methods of euthanasia assessed by infrared thermography. Animals.

[31] Lofgren J.L., Foley P.L., Golledge H.D.R., Suckow M.A., Hankenson R., Wilson R., Foley P.L. (2020). Anesthesia, analgesia, and euthanasia. The Laboratory Rat.

[32] Reimer J.N., Schuster C.J., Knight C.G., Pang D.S.J., Leung V.S.Y. (2020). Intraperitoneal injection of sodium pentobarbital has the potential to elicit pain in adult rats (*Rattus norvegicus*). PLoS ONE.

[33] American Veterinary Medical Association (AVMA) (2020). AVMA Guidelines for the Euthanasia of Animals.

[34] Risling T.E., Caulkett N.A., Florence D. (2012). Open-drop anesthesia for small laboratory animals. Can. Vet. J. Rev. Vet. Can..

[35] de Brito C.F., Evangelista A.A., Felippe R.M., Cascabulho C., Fragoso V.M., de Oliveira G.M. (2020). Laboratory Mice Euthanasia: Speed Death and Animal Welfare. Am. J. Biomed. Sci. Res..

[36] Ko M.J., Mulia G.E., van Rijn R.M. (2019). Commonly Used Anesthesia/Euthanasia Methods for Brain Collection Differentially Impact MAPK Activity in Male and Female C57BL/6 Mice. Front. Cell. Neurosci..

[37] Whittaker A.L., Leach M.C., Preston F.L., Lymn K.A., Howarth G.S. (2016). Effects of acute chemotherapy-induced mucositis on spontaneous behaviour and the grimace scale in laboratory rats. Lab. Anim..

[38] Diario Oficial de la Federación Normal Oficial Mexicana NOM-062-ZOO-1999, Especificaciones Técnicas para la Producción, Cuidado y Uso de los Animales de Laboratorio. https://www.gob.mx/cms/uploads/attachment/file/203498/NOM-062-ZOO-1999_220801.pdf.

[39] NOM-062-ZOO-1999 S. Especificaciones Técnicas para la producción, Cuidado y uso de los Animales de Laboratorio. http://publico.senasica.gob.mx/?doc=743.

[40] Oliver V., De Rantere D., Ritchie R., Chisholm J., Hecker K.G., Pang D.S.J. (2014). Psychometric Assessment of the Rat Grimace Scale and Development of an Analgesic Intervention Score. PLoS ONE.

[41] McLennan K.M., Miller A.L., Dalla Costa E., Stucke D., Corke M.J., Broom D.M., Leach M.C. (2019). Conceptual and methodological issues relating to pain assessment in mammals: The development and utilisation of pain facial expression scales. Appl. Anim. Behav. Sci..

[42] Kagias K., Nehammer C., Pocock R. (2012). Neuronal Responses to Physiological Stress. Front. Genet..

[43] Abdallah C.G., Geha P. (2017). Chronic Pain and Chronic Stress: Two Sides of the Same Coin?. Chronic Stress..

[44] Ballotta D., Lui F., Porro C.A., Nichelli P.F., Benuzzi F. (2018). Modulation of neural circuits underlying temporal production by facial expressions of pain. PLoS ONE.

[45] Ahmad A.H., Zakaria R. (2015). Pain in Times of Stress. Malays. J. Med. Sci..

[46] Avona A., Mason B.N., Lackovic J., Wajahat N., Motina M., Quigley L., Burgos-Vega C., Moldovan Loomis C., Garcia-Martinez L.F., Akopian A.N. (2020). Repetitive stress in mice causes migraine-like behaviors and calcitonin gene-related peptide-dependent hyperalgesic priming to a migraine trigger. Pain.

[47] Svendsen O., Kok L., Lauritzenã B. (2007). Nociception after intraperitoneal injection of a sodium pentobarbitone formulation with and without lidocaine in rats quantified by expression of neuronal c-fos in the spinal cord—A preliminary study. Lab. Anim..

[48] Laferriere C.A., Pang D.S. (2020). Review of Intraperitoneal Injection of Sodium Pentobarbital as a Method of Euthanasia in Laboratory Rodents. J. Am. Assoc. Lab. Anim. Sci..

[49] Shimizu S., Hedrich H.J., Bullock G. (2004). Routes of administration. The Laboratory Mouse.

[50] Boivin G.P., Bottomley M.A., Schiml P.A., Goss L., Grobe N. (2017). Physiologic, behavioral, and histologic responses to various euthanasia methods in C57BL76NTac male mice. J. Am. Assoc. Lab. Anim. Sci..

[51] Mion G., Villevieille T. (2013). Ketamine Pharmacology: An Update (Pharmacodynamics and Molecular Aspects, Recent Findings). CNS Neurosci. Ther..

[52] Sun F., Wright D., Pinson D.M. (2003). Comparison of ketamine versus combination of ketamine and medetomidine in injectable anesthetic protocols: Chemical immobilization in macaques and tissue reaction in rats. J. Am. Assoc. Lab. Anim. Sci..

[53] Hohlbaum K., Bert B., Dietze S., Palme R., Fink H., Thöne-Reineke C. (2018). Impact of repeated anesthesia with ketamine and xylazine on the well-being of C57BL/6JRj mice. PLoS ONE.

[54] Kash S.F., Tecott L.H., Hodge C., Baekkeskov S. (1999). Increased anxiety and altered responses to anxiolytics in mice deficient in the 65-kDa isoform of glutamic acid decarboxylase. Proc. Natl. Acad. Sci. USA.

[55] Kokkinou M., Ashok A.H., Howes O.D. (2018). The effects of ketamine on dopaminergic function: Meta-analysis and review of the implications for neuropsychiatric disorders. Mol. Psychiatry.

[56] Baek J.M., Kwak S.C., Kim J.-Y., Ahn S.-J., Jun H.Y., Yoon K.-H., Lee M.S., Oh J. (2015). Evaluation of a novel technique for intraperitoneal injections in mice. Lab Anim..

[57] Clarkson J.M., Martin J.E., McKeegan D.E.F. (2022). A review of methods used to kill laboratory rodents: Issues and opportunities. Lab. Anim..

[58] Mikeska J.A., Klemm W.R. (1975). EEG evaluation of humaneness of asphyxia and decapitation euthanasia of the laboratory rat. Lab. Anim. Sci..

[59] Cartner S.C., Barlow S.C., Ness T.J. (2007). Loss of cortical function in mice after decapitation, cervical dislocation, potassium chloride injection, and CO_2_ inhalation. Comp. Med..

[60] Pierozan P., Jernerén F., Ransome Y., Karlsson O. (2017). The Choice of Euthanasia Method Affects Metabolic Serum Biomarkers. Basic Clin. Pharmacol. Toxicol..

[61] Singh P., Kongara K., Harding D., Ward N., Dukkipati V.S.R., Johnson C., Chambers P. (2018). Comparison of electroencephalographic changes in response to acute electrical and thermal stimuli with the tail flick and hot plate test in rats administered with opiorphin. BMC Neurol..

[62] Mota-Rojas D., Olmos-Hernández A., Verduzco-Mendoza A., Hernández E., Martínez-Burnes J., Whiattaker A.L. (2020). The utility of grimace scales for practical pain assessment in laboratory animals. Animals.

[63] Améndola L., Weary D.M. (2020). Understanding rat emotional responses to CO_2_. Transl. Psychiatry.

[64] Thürauf N., Friedel I., Hummel C., Kobal G. (1991). The mucosal potential elicited by noxious chemical stimuli with CO_2_ in rats: Is it a peripheral nociceptive event?. Neurosci. Lett..

[65] Conlee K.M., Stephens M.L., Rowan A.N., King L.A. (2005). Carbon dioxide for euthanasia: Concerns regarding pain and distress, with special reference to mice and rats. Lab. Anim..

[66] Turner P.V., Hickman D.L., van Luijk J., Ritskes-Hoitinga M., Sargeant J.M., Kurosawa T.M., Agui T., Baumans V., Choi W.S., Choi Y.-K. (2020). Welfare Impact of Carbon Dioxide Euthanasia on Laboratory Mice and Rats: A Systematic Review. Front. Vet. Sci..

[67] Leach M.C., Bowell V.A., Allan T.F., Morton D.B. (2002). Degrees of aversion shown by rats and mice to different concentrations of inhalational anaesthetics. Vet. Rec..

[68] Burkholder T.H., Niel L., Weed J.L., Brinster L.R., Bacher J.D., Foltz C.J. (2010). Comparison of carbon dioxide and argon euthanasia: Effects on behavior, heart rate, and respiratory lesions in rats. J. Am. Assoc. Lab. Anim. Sci..

[69] Valentim A.M., Guedes S.R., Pereira A.M., Antunes L.M. (2016). Euthanasia using gaseous agents in laboratory rodents. Lab. Anim..

[70] Miller A., Golledge H., Leach M. (2016). The influence of isoflurane anaesthesia on the rat grimace scale. PLoS ONE.

[71] Miller A., Kitson G., Skalkoyannis B., Leach M. (2015). The effect of isoflurane anaesthesia and buprenorphine on the mouse grimace scale and behaviour in CBA and DBA/2 mice. Appl. Anim. Behav. Sci..

[72] Hohlbaum K., Bert B., Dietze S., Palme R., Fink H., Thöne-Reineke C. (2017). Severity classification of repeated isoflurane anesthesia in C57BL/6JRj mice—Assessing the degree of distress. PLoS ONE.

[73] Wong D., Makowska I.J., Weary D.M. (2013). Rat aversion to isoflurane versus carbon dioxide. Biol. Lett..

[74] Bodnar M.J., Ratuski A.S., Weary D.M. (2023). Mouse isoflurane anesthesia using the drop method. Lab. Anim..

[75] Sio L.C.O., Varbanova M., Bautista A., Rajendram R., Patel V.B., Preedy V.R., Martin C.R. (2022). Isoflurane: Mechanisms and applications. Treatments, Mechanisms, and Adverse Reactions of Anesthetics and Analgesics.

[76] Pai A., Heining M. (2007). Ketamine. Contin. Educ. Anaesth. Crit. Care Pain.

[77] Valentine H., Williams W.O., Maurer K.J. (2012). Sedation or inhalant anesthesia before euthanasia with CO_2_ does not reduce behavioral or physiologic signs of pain and stress in mice. J. Am. Assoc. Lab. Anim. Sci..

[78] Zhang Y., Ye F., Zhang T., Lv S., Zhou L., Du D., Lin H., Guo F., Luo C., Zhu S. (2021). Structural basis of ketamine action on human NMDA receptors. Nature.

[79] Hashimoto K. (2019). Rapid-acting antidepressant ketamine, its metabolites and other candidates: A historical overview and future perspective. Psychiatry Clin. Neurosci..

[80] Larson C.M., Wilcox G.L., Fairbanks C.A. (2019). The Study of Pain in Rats and Mice. Comp. Med..

[81] Aulehner K., Leenaars C., Buchecker V., Stirling H., Schönhoff K., King H., Häger C., Koska I., Jirkof P., Bleich A. (2022). Grimace scale, burrowing, and nest building for the assessment of post-surgical pain in mice and rats—A systematic review. Front. Vet. Sci..

[82] Brown E.N., Purdon P.L., Van Dort C.J. (2011). General Anesthesia and Altered States of Arousal: A Systems Neuroscience Analysis. Annu. Rev. Neurosci..

[83] Tomberg C., Petagna M., de Selliers de Moranville L.-A. (2023). Horses (Equus caballus) facial micro-expressions: Insight into discreet social information. Sci. Rep..

[84] Rodriguez-Sanchez R., Barnaby E., Améndola L., Hea S.-Y., Smith B., Webster J., Zobel G. (2021). Voluntary Oral Ingestion of a Sedative Prior to Euthanasia with CO_2_: Behavioural Responses of Mice. Animals.

[85] Dudley E.S., Boivin G.P. (2018). Evaluation of a Commercially Available Euthanasia Solution as a Voluntarily Ingested Euthanasia Agent in Laboratory Mice. J. Am. Assoc. Lab. Anim. Sci..

[86] Winter A., Ahlbrand R., Naik D., Sah R. (2017). Differential behavioral sensitivity to carbon dioxide (CO_2_) inhalation in rats. Neuroscience.

